# Decreased Circulating Endothelial Progenitor Cell Levels and Function in Patients with Nonalcoholic Fatty Liver Disease

**DOI:** 10.1371/journal.pone.0031799

**Published:** 2012-02-16

**Authors:** Chia-Hung Chiang, Po-Hsun Huang, Fa-Po Chung, Zu-Yin Chen, Hsin-Bang Leu, Chin-Chou Huang, Tao-Cheng Wu, Jaw-Wen Chen, Shing-Jong Lin

**Affiliations:** 1 Division of Cardiology, Taipei Veterans General Hospital, Taipei, Taiwan; 2 Department of Medical Research and Education, Taipei Veterans General Hospital, Taipei, Taiwan; 3 Healthcare and Management Center, Taipei Veterans General Hospital, Taipei, Taiwan; 4 Institute of Clinical Medicine, National Yang-Ming University, Taipei, Taiwan; 5 Cardiovascular Research Center, National Yang-Ming University, Taipei, Taiwan; 6 Institute and Department of Pharmacology, National Yang-Ming University, Taipei, Taiwan; University of Tor Vergata, Italy

## Abstract

**Objectives:**

Nonalcoholic fatty liver disease (NAFLD) is associated with advanced atherosclerosis and a higher risk of cardiovascular disease. Increasing evidence suggests that injured endothelial monolayer is regenerated by circulating bone marrow derived-endothelial progenitor cells (EPCs), and levels of circulating EPCs reflect vascular repair capacity. However, the relation between NAFLD and EPC remains unclear. Here, we tested the hypothesis that patients with nonalcoholic fatty liver disease (NAFLD) might have decreased endothelial progenitor cell (EPC) levels and attenuated EPC function.

**Methods and Results:**

A total of 312 consecutive patients undergoing elective coronary angiography because of suspected coronary artery disease were screened and received examinations of abdominal ultrasonography between July 2009 and November 2010. Finally, 34 patients with an ultrasonographic diagnosis of NAFLD, and 68 age- and sex-matched controls without NAFLD were enrolled. Flow cytometry with quantification of EPC markers (defined as CD34^+^, CD34^+^KDR^+^, and CD34^+^KDR^+^CD133^+^) in peripheral blood samples was used to assess circulating EPC numbers. The adhesive function, and migration, and tube formation capacities of EPCs were also determined in NAFLD patients and controls. Patients with NAFLD had a significantly higher incidence of metabolic syndrome, previous myocardial infarction, hyperuricemia, and higher waist circumference, body mass index, fasting glucose and triglyceride levels. In addition, patients with NAFLD had significantly decreased circulating EPC levels (all P<0.05), attenuated EPC functions, and enhanced systemic inflammation compared to controls. Multivariate logistic regression analysis showed that circulating EPC level (CD34^+^KDR^+^ [cells/10^5^ events]) was an independent reverse predictor of NAFLD (Odds ratio: 0.78; 95% confidence interval: 0.69–0.89, P<0.001).

**Conclusions:**

NAFLD patients have decreased circulating EPC numbers and functions than those without NAFLD, which may be one of the mechanisms to explain atherosclerotic disease progression and enhanced cardiovascular risk in patients with NAFLD.

## Introduction

Nonalcoholic fatty liver disease (NAFLD) is a highly prevalent condition characterized by fatty infiltration of liver cells. The clinical manifestations of NAFLD resemble those of alcohol-induced liver injury, but NAFLD occurs in patients who do not abuse alcohol [1]. The prevalence of NAFLD is generally between 10% and 40% in various populations, and it is also the most common cause of abnormal results in liver function tests [2]–[4]. There is growing evidence that NAFLD, a hepatic manifestation of the metabolic syndrome [5], is strongly associated with obesity, insulin resistance, enhanced systemic inflammation, and advanced atherosclerosis, independent of shared cardiometabolic risk factors [6], [7]. Previous studies have suggested that non-obese subjects with NAFLD have a significantly increased cardiovascular disease risk [8], [9]. However, the pathophysiologic mechanisms underlying the evolution from NAFLD to atherosclerosis and cardiovascular events remain to be determined.

Convincing evidence indicates that atherosclerosis is associated with endothelial dysfunction at the early stage of the disease process [10]. Intact endothelium and maintenance of endothelial integrity play a pivotal role in preventing the development of atherosclerotic vascular disease [11]. Recent insight suggests that the injured endothelial monolayer is regenerated by circulating bone marrow derived-endothelial progenitor cells (EPCs) [12], and levels of circulating EPCs reflect endothelial repair capacity [13]. An altered status of circulating EPCs represents a marker of endothelial dysfunction and vascular health, and the level of circulating EPCs could be used as a surrogate index of cumulative cardiovascular risk [14]. Circulating EPC number has also been reported to inversely correlate with presence of risk factors of coronary artery disease [14]–[16]. Furthermore, a reduced number of circulating EPCs independently predicts atherosclerotic disease progression and future cardiovascular events [17]. Clinical studies have indicated that NAFLD is associated with arterial stiffness and endothelial dysfunction [18], [19]. However, no previous report has mentioned the role of circulating EPCs in patients with NAFLD. In this study, we tested the hypothesis that decreased circulating EPC levels and function might be associated with NAFLD and that this could be one mechanism to explain the higher risk of cardiovascular disease among NAFLD patients.

## Methods

### Study participants

We initially screened a total of 312 consecutive patients, who were admitted to Taipei Veterans General Hospital between July 2009 and November 2010 to undergo elective coronary angiography because of suspected coronary artery disease. Subjects were excluded from the study on the basis of the following criteria: (1) presence of serological markers of hepatitis B virus (hepatitis B surface antigem and anti-HBs antibody) and hepatitis C virus infection (anti-HCV antibody); (2) presence of autoimmune liver disease or alcoholic liver disease (alcohol intake more than 20 g per day by using a questionnaire); (3) presence of malignant diseases, or (4) clinical evidence of unstable angina, myocardial infarction, congestive heart failure, valvular heart disease, inflammatory disease, or thyroid dysfunction. The presence of NAFLD was identified by ultrasonographic findings. Abdominal ultrasonography was performed before examination of the coronary angiography by two of four experienced gastroenterologists, who were blinded to the clinical presentation and laboratory findings. The severity of fatty liver was graded as follows: (1) mild, defined as a slight, diffuse increase in liver echogenicity in the hepatic parenchyma with normal visualization of the diaphragm and the portal veins; (2) moderate, defined as a moderate, diffuse increase in liver echogenicity with slightly impaired visualization of the diaphragm and the portal veins; (3) severe, defined as a marked increase in liver echogenicity with poor or no visualization of the diaphragm and the portal veins [20]. To decrease inter-observer variation, the ultrasonographic severity of NAFLD measured by individual gastroenterologist was randomly monitored and reviewed by a senior gastroenterologist. In case of disagreement, the opinion of the third observer was obtained, and the final decision was made by consensus.

On the basis of these screening criteria, 34 patients with a diagnosis of NAFLD, as the study group, and 68 age- and gender-matched patients without NAFLD, as controls, were enrolled in this study. Medical history, including information about conventional cardiovascular risk factors (smoking, hypertension, diabetes mellitus, hyperlipidemia, peripheral artery disease, and chronic kidney disease), previous cardiovascular events (myocardial infarction and cerebrovascular disease), and current drug treatment was obtained during a personal interview and from medical files. Coronary artery disease was defined as having ≧ 50% stenosis of one or more of the major coronary arteries on the basis of the results of coronary angiography. Diagnosis of metabolic syndrome was based on the definition proposed by the National Cholesterol Education Program Adult Treatment Panel III (NCEP ATP III). The waist circumference cut-off point in the ATP III criteria was revised as suggested by the 2000 World Health Organization (WHO) Asia-Pacific Guidelines, because the absolute risk of diabetes and cardiovascular disease is higher in Asians, who are less obese [21]. Presence of any 3 of following criteria was considered grounds for diagnosis of metabolic syndrome: (1) fasting glucose ≥100 mg/dL or treated for diabetes; (2) waist circumference >90 cm in men and >80 cm in women; (3) blood pressure ≥130/≥85 mmHg or pharmacological treatment for high blood pressure; (4) triglyceride levels ≥150 mg/dL or current use of fibrates, and (5) high-density lipoprotein cholesterol (HDL-C) <40 mg/dL in men and <50 mg/dL in women.

### Ethics statements

This study was approved by Taipei Veterans General Hospital research ethics committee. All patients gave written informed consent and research was conducted according to the principles expressed in the Declaration of Helsinki.

### Laboratory investigations

Blood pressure was recorded as the average of 3 different measurements taken after 15-minute resting periods. Body mass index (BMI) was calculated by dividing the weight of the patient in kilograms by the square of the height in meters. Waist circumference was measured in a standard position at the level of the umbilicus. Venous blood was drawn in the morning after an overnight fast. Plasma liver function tests and other biochemical blood measurements, including assessments of fasting blood glucose, uric acid, creatinine, total cholesterol, high-density lipoprotein cholesterol (HDL-C), gamma-glutamyl transferase (γGT), and triglyceride levels were performed by standard laboratory procedures. All participants had negative results in serological tests for hepatitis B or C. High sensitivity C-reactive protein (hsCRP) levels in plasma were assessed using the latex-enhanced immunonephelometric assay (Dade Behring, Marburg, Germany) [22]. Plasma asymmetric dimethylarginine (ADMA) levels were determined using a quantitative sandwich enzyme immunoassay technique with an ADMA enzyme-linked immunosorbent assay kit (DLD Diagnostika GmbH, Hamburg, Germany) [23]. The intra-assay and inter-assay variation coefficients were not more than 4% and 8%, respectively [24].

### Assay of circulating EPCs

**Figure 1:**
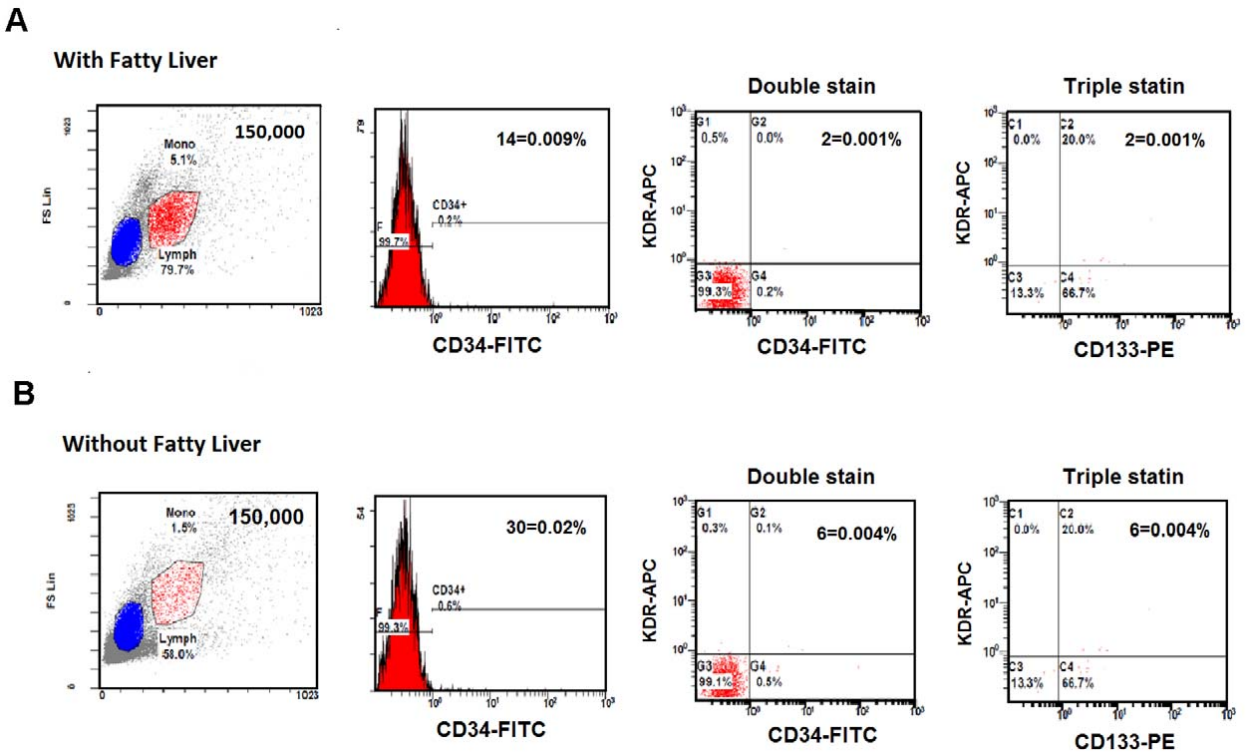
Representative flow cytometry analysis for quantifying the number of circulating endothelial progenitor cells (EPCs). Upper left shows mononuclear cells (MNCs) were gated by forward/sideward scatter (FSC/SSC) in patients with nonalcoholic fatty liver disease (NAFLD) (A) and without NAFLD (B). The numbers of circulating EPCs were defined as CD34^+^, CD34^+^KDR^+^, and CD34^+^KDR^+^CD133^+^, respectively.

Assessment of the circulating EPCs by flow cytometry was performed by the researchers masked to the clinical data [25]. A volume of 1000-µL peripheral blood was incubated for 30 minutes in the dark with monoclonal antibodies against human KDR (R&D, Minneapolis, MN, USA) followed by Allophycocyanin(APC)-conjugated secondary antibody, with the fluorescein isothiocyanate (FITC)-labeled monoclonal antibodies against human CD45 (Becton Dickinson, Franklin Lakes, NJ, USA), with the PE-conjugated monoclonal antibody against human CD133 (Miltenyi Biotec, Germany), and with FITC-conjugated monoclonal antibodies against human CD34 (Becton Dickinson Pharmingen, USA). After incubation, cells were lysed, washed with phosphate-buffered saline (PBS), and fixed in 2% paraformaldehyde before analysis. Each analysis included 150,000 events. As shown in Figure 1, the numbers of circulating EPCs were gated with monocytes and defined as CD34^+^CD45^low^, CD34^+^KDR^+^CD45^low^, and CD34^+^KDR^+^CD133^+^CD45^low^, respectively. The number of CD34^+^, CD34^+^KDR^+^, and CD34^+^KDR^+^CD133^+^ cells was normalized and expressed percentage (%) and cells per 1×10^5^ events. To assess the reproducibility of EPC measurements, circulating EPCs were measured from 2 separate blood samples in 10 subjects, and there was a strong correlation between the two measurements (r = 0.90, *P*<0.001).

### Human early and late EPC cultivation

Peripheral blood samples (20 ml) were obtained from study populations, and total mononuclear cells (MNCs) were isolated by density gradient centrifugation with Histopaque-1077 (Sigma, St. Louis, MO, USA) [23]. Briefly, MNCs (5×10^6^) were plated in 2 ml endothelial growth medium (EGM-2 MV Cambrex, East Rutherford, NJ, USA) on fibronectin-coated 6-well plates. After 4 days of culturing, the medium was changed and nonadherent cells were removed; attached early EPCs appeared elongated with a spindle shape. A certain number of MNCs were allowed to grow into colonies of late EPCs, which emerged 2–4 weeks after the start of the MNC culture. The late EPCs exhibited a “cobblestone” morphology and monolayer growth pattern typical of mature endothelial cells at confluence. Both early and late EPCs were collected and used for the functional assays in this study. The characteristics and phenotype of the early and late EPC were defined by flow cytometry (Figure S1).

### EPC characterization

The early EPCs were characterized as adherent cells double positive for acetylated low-density lipoprotein uptake and lectin binding by direct fluorescent staining as previously described [26]. The early and late EPCs were characterized by immunofluorescence staining for the expression of VE-cadherin, platelet/endothelial cell adhesion molecule-1 (PECAM-1) (CD-31), CD34, KDR (VEGFR-2), and AC133 (Santa Cruz Biotechnology, Inc., CA, USA). The fluorescent images were recorded under a laser scanning confocal microscope.

### Fibronectin adhesion assay of EPCs

Early EPCs (day 7) from 16 subjects (8 NAFLD patients and 8 controls) were washed with phosphate-buffered saline and gently detached with 0.5 mmol/L EDTA in phosphate-buffered saline. The basic characteristics of those 2 groups were similar (Table S1). After centrifugation and re-suspension in basal medium with 5% fetal bovine serum, identical cells were placed on a fibronectin-coated 6-well plate and incubated for 30 min at 37°C. Gentle washing with phosphate-buffered saline was performed 3 times after adhesion for 30 minutes, and adherent cells were counted by independent blinded investigators [26]. Phenotyping of the endothelial characteristics of adherent cells by indirect immunostaining was performed with FITC-labeled lectin from *Ulex europaeus* (UEA-1). Briefly, the adherent cells were fixed in 2% paraformaldehyde and incubated with 10 µg/mL FITC-labeled UEA-1 (Sigma) as previously described [26].

### EPC migration test

The migratory function of late EPCs was evaluated by a modified Boyden chamber assay (Transwell, Coster, San Diego, CA, USA) [23]. Briefly, isolated EPCs were detached as described above with trypsin/EDTA and then 4×10^4^ late EPCs were placed in the upper chambers of 24-well Transwell plates with polycarbonate membrane (8-µm pores) with serum-free endothelial growth medium; VEGF (50 ng/ml) in medium was placed in the lower chamber. After incubation for 24 hours, the membrane was washed briefly with PBS and fixed with 4% paraformaldehyde. The membrane was then stained using hematoxylin solution and carefully removed. The magnitude of migration of the late EPCs was evaluated by counting the migrated cells in six random high-power (×100) microscopic fields.

### EPC tube formation assay

An EPC tube formation assay was performed using the In Vitro Angiogenesis Assay Kit (Chemicon) [23]. ECMatrix gel solution was thawed overnight at 4°C, mixed with ECMatrix diluent buffer, and placed in a 96-well plate for 1 h at 37°C to allow the matrix solution to solidify. Late EPCs were harvested with trypsin/EDTA, as described above, and 1×10^4^ EPCs were placed onto a matrix with EGM-2 MV medium and incubated at 37°C for 16 h. Tubule formation was inspected with an inverted light microscope (100×). Six representative fields were used to determine the average of the total area of complete tubes formed by cells using the computer software, Image-Pro Plus.

### Statistical analysis

Data were expressed as the mean ± standard deviation (SD) for numeric variables and as the number (percent) for categorical variables. Comparisons of continuous variables between groups were performed by Student's *t* test and one-way ANOVA. Subgroup comparisons of categorical variables were assessed by Chi-square or Fisher's exact test. To examine the effects of various factors on NAFLD, several factors and EPC levels were considered as confounders for univariate and multivariate logistic regression analysis separately. Data were analyzed using SPSS software (version 17, SPSS, Chicago, Illinois, USA). A P value of <0.05 was considered to indicate statistical significance.

## Results

### Clinical and laboratory data

The mean age of the 102 study patients (48 males, 47%) was 70±14 years. The patients with NAFLD in the study group and those without NAFLD in the control group were matched for age and gender. The baseline characteristics of all study subjects are presented in Table 1. No significant differences were noted between the 2 groups, including age, gender, hypertension, type 2 diabetes mellitus, coronary artery disease, peripheral artery disease, chronic kidney disease, hyperlipidemia, smoking, atrial fibrillation, or previous history of cerebrovascular disease. However, NAFLD patients had a higher incidence of metabolic syndrome, hyperuricemia, and previous myocardial infarction. There were no significant differences between the 2 groups in terms of currently used medications, including antiplatelet agents, angiotensin-converting enzyme inhibitors, angiotensin receptor blockers, calcium channel blockers, beta blockers, diuretics, peroxisome proliferator-activated receptor gamma agonists, statins, nitrates, metformin, and insulin. In addition, patients with NAFLD had significantly higher waist circumference and BMI values, as well as increased plasma uric acid and lower HDL-C levels than those without NAFLD (Table 2).

**Table 1:** Baseline characteristics of study subjects.

	No fatty liver	Fatty liver	P value
	(n = 68)	(n = 34)	
Age (years)	70±13	71±15	0.191
Male, n (%)	32 (47)	16 (47)	1.000
Hypertension, n (%)	56 (82)	29 (85)	0.925
Type 2 diabetes mellitus, n (%)	26 (38)	16 (47)	0.522
Metabolic syndrome, n (%)	28 (41)	24 (71)	0.010
Coronary artery disease, n (%)	39 (57)	25 (74)	0.169
Peripheral artery disease, n (%)	15 (22)	12 (35)	0.234
Chronic kidney disease, n (%)	27 (40)	17 (50)	0.364
Hyperlipidemia, n (%)	37 (54)	21 (62)	0.621
Current smoker, n (%)	12 (18)	9 (27)	0.311
Previous myocardial infarction, n (%)	14 (21)	15 (44)	0.024
Previous cerebrovascular disease, n (%)	10 (15)	6 (18)	0.923
Atrial fibrillation, n (%)	11 (16)	7 (21)	0.783
Hyperuricemia, n (%)	18 (27)	26 (77)	<0.001

Values are mean ± standard deviation (SD) or number (%).

**Table 2:** Metabolic profiles and medications of study subjects.

	No fatty liver(n = 68)	Fatty liver(n = 34)	P value
Waist circumference (cm)	84.4±9.3	89.8±7.8	0.005
BMI (kg/m^2^)	24.6±4.0	26.7±5.3	0.029
Cholesterol (mg/dL)	168±48	175±38	0.406
LDL-C (mg/dL)	98±45	106±32	0.366
HDL-C (mg/dL)	47±13	41±12	0.048
Triglyceride (mg/dL)	119±62	143±90	0.115
Creatinine (mg/dL)	1.8±2.3	1.9±2.0	0.972
Total bilirubin (mg/dL)	0.5±0.2	0.6±0.4	0.291
ALT (U/L)	23±23	35±37	0.055
γGT (U/L)	39±65	47±60	0.723
Uric acid (mg/dL)	5.9±1.8	7.9±2.5	<0.001
Fasting glucose (mg/dL)	135±62	136±59	0.933
HbA1c (%)	6.9±0.6	7.1±0.7	0.370
Medication			
Aspirin, n (%)	43 (63)	23 (68)	0.826
Clopidogrel, n (%)	33 (49)	18 (53)	0.834
ACEI, n (%)	9 (13)	3 (9)	0.744
ARB, n (%)	25 (37)	12 (35)	1.000
CCB, n (%)	31 (46)	15 (44)	1.000
Beta blockers, n (%)	27 (40)	15 (44)	0.831
Diuretics, n (%)	23 (34)	13 (38)	0.826
PPAR-γ agonists, n (%)	10 (15)	8 (24)	0.409
Statins, n (%)	34 (50)	16 (47)	0.944
Nitrates, n (%)	32 (47)	20 (59)	0.363
Metformin, n (%)	13 (19)	8 (24)	0.612
Insulin , n (%)	7 (10)	5 (15)	0.528

Values are mean ± standard deviation (SD) or number (%).

HbA1c levels of type 2 diabetes patients.

BMI: body mass index; LDL-C: low-density lipoprotein cholesterol; HDL-C: high-density lipoprotein cholesterol; ALT: alanine aminotransferase; HbA1c: Hemoglobin A1c; γGT: gamma-glutamyl transferase; ACEI: angiotensin-converting enzyme inhibitor; ARB: angiotensin receptor blocker; CCB: calcium channel blocker; PPAR-γ agonists: peroxisome proliferator-activated receptor gamma agonists.

### Circulating EPC levels

**Figure 2:**
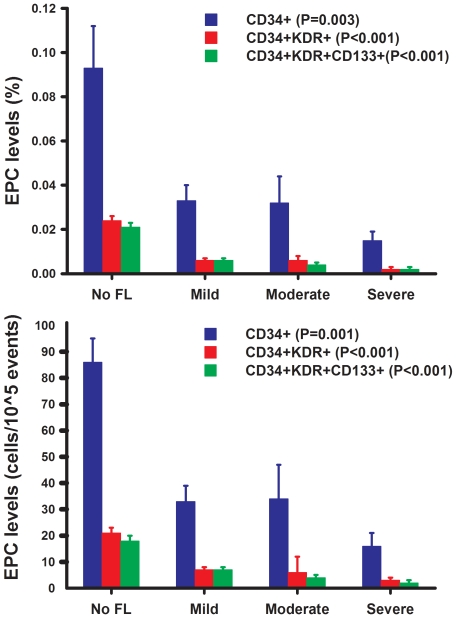
The association between EPC levels (% and cells/10^5^ events) and the severity of non-alcoholic fatty liver disease (values presented as means ± standard error; FL, fatty liver; Mild, mild fatty liver; Moderate, moderate fatty liver; Severe, severe fatty liver).

As shown in Table 3, NAFLD patients had significantly decreased levels of circulating EPCs (CD34^+^, CD34^+^KDR^+^, and CD34^+^KDR^+^CD133^+^, all P<0.05). For further analysis, subjects with NAFLD were divided into 3 groups according to the severity of fatty liver in ultrasonographic analysis: group 1, mild fatty liver (n = 17); group 2, moderate fatty liver (n = 10), and group 3, severe fatty liver (n = 7). Circulating EPC numbers were negatively associated with the severity of fatty liver (No fatty liver vs. mild vs. moderate vs. severe fatty liver, [mean ± SD]: CD34^+^: 0.093%±0.093% vs. 0.033%±0.027% vs. 0.032%±0.039% vs. 0.015%±0.011%, P = 0.003; CD34^+^KDR^+^: 0.024%±0.016% vs. 0.006%±0.006% vs. 0.006%±0.006% vs. 0.002%±0.001%, P<0.001; CD34^+^KDR^+^CD133^+^: 0.021%±0.016% vs. 0.006%±0.005% vs. 0.004%±0.004% vs. 0.002%±0.002%, P<0.001; Figure 2). The characteristics of subjects with mild, moderate, and severe fatty liver were shown in Table S2 in the supplemental material.

**Table 3:** Comparison of the levels of circulating endothelial progenitor cells, inflammatory markers, and ADMA in fatty liver patients versus controls.

	No fatty liver(n = 68)	Fatty liver(n = 34)	P value
EPC levels (%)			
CD34^+^	0.093±0.093	0.029±0.029	0.003
CD34^+^KDR^+^	0.024±0.016	0.005±0.005	<0.001
CD34^+^KDR^+^CD133^+^	0.021±0.016	0.005±0.004	<0.001
EPC levels (cells/10^5^ events)			
CD34^+^	86±74	30±30	0.017
CD34^+^KDR^+^	21±15	6±6	<0.001
CD34^+^KDR^+^CD133^+^	17±14	5±5	<0.001
hsCRP (mg/L)	0.96±0.96	1.93±1.70	0.013
ADMA (µmol/L)	0.66±0.49	0.78±0.42	0.269

Values are mean ± standard deviation (SD).

EPC: endothelial progenitor cell; hsCRP: high sensitivity C-reactive protein; ADMA: asymmetric dimethylarginine.

### Characterization of human EPC and functions

**Figure 3:**
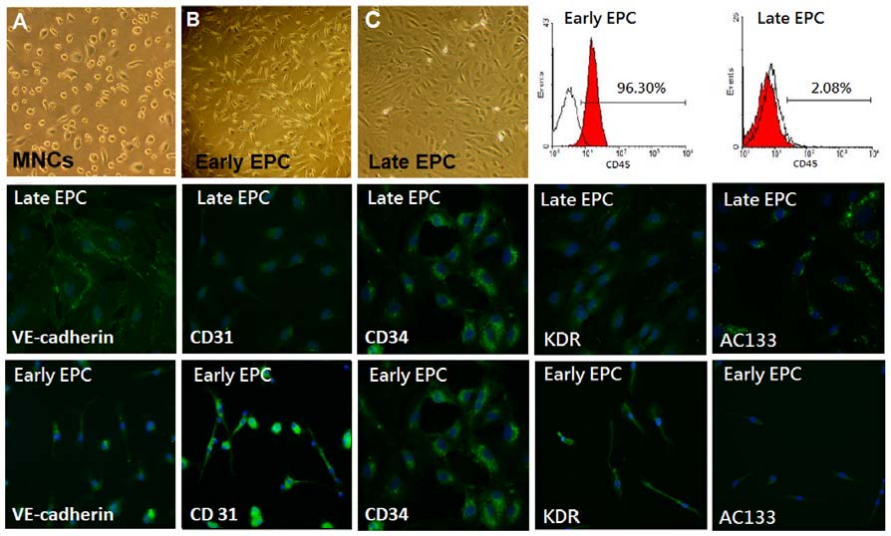
Morphology and characterization of human endothelial progenitor cells (EPCs) from peripheral blood. Peripheral blood mononuclear cells (MNCs) were plated on a fibronectin-coated culture dish on the first day (**A**). Four days after plating, adherent early EPCs with a spindle shape were shown (**B**). Three weeks after plating, late EPCs with a cobblestone-like morphology were selected, reseeded, and grown to confluence (**C**). Early and late EPC characterization were performed by flow cytometry analysis (CD45) and immunohistochemical staining. Most of the EPC expressed endothelial and hematopoietic stem cell markers, VE-cadherin, PECAM-1 (CD31), CD34, KDR, and AC133 (Figure 3), which are considered critical markers of EPCs. Cells were counterstained with 4′,6-diamidino-2-phenylindole (DAPI) for the nuclei (blue).

**Figure 4:**
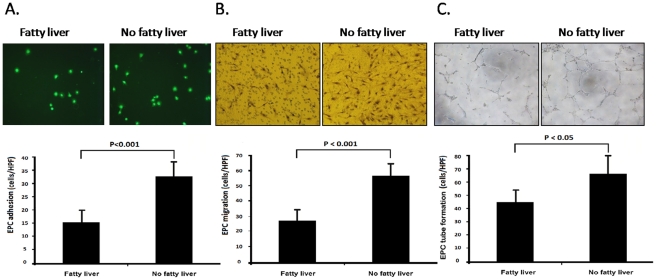
Comparison of the EPC adhesive function (A), migration (B), and tube formation capacities (C) in subjects with or without fatty liver (values presented as means ± SD; HPF: high-power field; *P<0.05).

Early and late EPCs were isolated from peripheral blood MNCs of healthy subjects as previously described [23], [26]. The peripheral blood MNCs that initially seeded on fibronectin-coated wells were round in shape (Figure 3A). After the medium was changed on day 4, attached early EPCs appeared to be elongated with a spindle shape (Figure 3B). Late EPCs with a cobblestone-like morphology similar to mature endothelial cells were grown to confluence (Figure 3C). Early and late EPC characterization were performed by flow cytometry analysis (CD45) and immunohistochemical staining, and most of the EPC expressed endothelial and hematopoietic stem cell markers, VE-cadherin, PECAM-1 (CD31), CD34, KDR, and AC133 (Figure 3), which are considered critical markers of EPCs, but early and late EPC markers are different in cell surface makers. (Figure S1) Patients with NAFLD showed attenuated EPC adhesive function in comparison to those without NAFLD (control vs. NAFLD, 32.6±6.3 vs. 15.4±5.7 cells/HPF, P<0.001; Figure 4A). Moreover, Patients with NAFLD had impaired EPC migration (control vs. NAFLD, 56.5±6.9 vs. 27.4±8.1 cells/HPF, P<0.001; Figure 4B) and tube formation capacity (control vs. NAFLD, 65.0±13.5 vs. 45.0±9.0 cells/HPF, P<0.05; Figure 4C) in comparison to those without NAFLD.

Patients with NAFLD had significantly higher plasma concentrations of hsCRP, a downstream marker of inflammation, indicating higher systemic inflammation than in the controls (control vs. NAFLD: 0.96±0.96 vs. 1.93±1.70 mg/L, P = 0.013). In addition, the plasma levels of ADMA were nonsignificantly enhanced in patients with NAFLD (control vs. NAFLD: 0.66±0.49 vs. 0.78±0.42 µmol/L, P = 0.269).

### Independent correlates of nonalcoholic fatty liver disease

In order to identify the independent predictors of NAFLD, univariate and multivariate logistic regression analyses were performed. As shown in Table 4, using univariate analysis, reduced circulating EPC level (CD34^+^KDR^+^ [cells/10^5^ events]), metabolic syndrome, uric acid, and hsCRP were found to be significant predictors of NAFLD. After adjustment for metabolic syndrome and uric acid levels, circulating EPC level was still an independent negative predictor of NAFLD (Odds ratio [OR]: 0.78; 95% confidence interval: 0.69–0.89, P<0.001). Although NAFLD is strongly associated with metabolic syndrome and diabetes mellitus, EPC level keeps an inverse association with NAFLD after adjustment for metabolic syndrome and diabetes mellitus (OR: 0.76; 95% confidence interval: 0.67–0.87, P<0.001). As smoking has an impact on EPC level, we excluded smokers and re-analyze whether EPC remained significantly decreased in patients with NAFLD. Totally, there were 56 controls and 25 fatty liver patients without previous smoking history. Among them, patients with NAFLD still had significantly lower EPC numbers compared to controls (P<0.001), and EPC level (CD34^+^KDR^+^ [cells/10^5^ events]) was still a negative predictor of fatty liver (OR: 0.79, 0.69–0.90, P = 0.001).

**Table 4:** Simple correlation and multivariate analysis of factors associated with nonalcoholic fatty liver disease.

	OR (95% CI)	P value
Univariate analysis		
CD34^+^KDR^+^ EPCs (cells/10^5^ events)	0.74 (0.65–0.85)	<0.001
Age	1.11 (0.81–2.57)	0.805
Male	1.13 (0.49–2.57)	0.778
Hypertension	1.23 (0.39–3.88)	0.728
Diabetes	1.44 (0.62–3.36)	0.391
Hyperlipidemia	1.29 (0.56–3.03)	0.555
Metabolic syndrome	3.90 (1.59–9.55)	0.003
Peripheral artery disease	1.91 (0.76–4.79)	0.169
Coronary artery disease	2.22 (0.90–5.52)	0.085
Chronic kidney disease	1.74 (0.75–4.05)	0.200
Current smoking	1.68 (0.63–4.50)	0.302
Uric acid (mg/dL)	1.61 (1.25–2.08)	<0.001
hsCRP (mg/L)	1.79 (1.07–2.97)	0.026
ADMA (µmol/L)	1.78 (0.61–5.18)	0.293
*Multivariate analysis		
CD34^+^KDR^+^ EPCs (cells/10^5^ events)	0.78 (0.69–0.89)	<0.001

OR: odds ratio; CI: confidence interval; EPC: endothelial progenitor cell; hsCRP: high sensitivity C-reactive protein; ADMA: asymmetric dimethylarginine.

* Multivariate analysis: adjusted for metabolic syndrome and uric acid levels.

## Discussion

To the best of our knowledge, this is the first study to show that patients with NAFLD have decreased circulating EPC numbers and impaired adhesive function and migration than those without NAFLD. Furthermore, patients with NAFLD have an enhanced inflammatory state, and the severity of NAFLD determined on the basis of ultrasonographic findings is negatively associated with circulating EPC levels. These findings suggest that attenuated endothelial repair capacity may contribute to atherosclerotic disease progression and increased risk of cardiovascular events in NAFLD patients.

NAFLD is a highly prevalent condition characterized by fatty infiltration of liver cells resembling that of alcohol-induced liver injury, but it occurs in patients who do not abuse alcohol [1]. It includes a spectrum of liver damage ranging from simple steatosis to nonalcoholic steatohepatitis (NASH), advanced fibrosis, and rarely, progression to cirrhosis. Because the patients with metabolic syndrome and those with NAFLD have similar clinical characteristics, an increased risk of cardiovascular disease is expected in NAFLD patients. Accumulating evidence suggests that cardiovascular mortality is increased in patients with a diagnosis of fatty liver of nonalcoholic or unspecified causes [27], [28]. In a community-based cohort study of 2,088 male workers, the presence of ultrasonographic evidence of NAFLD was independently associated with an increased prevalence of ischemic heart disease [29]. Moreover, in patients consecutively referred for elective coronary angiography, those with NAFLD have more severe coronary artery disease, which occurs independent of established conventional cardiovascular risk factors [30]. Clinical studies further suggest that NAFLD is a strong risk factor for endothelial dysfunction and carotid atherosclerosis beyond its association with metabolic syndrome [31]. However, the pathophysiologic mechanisms underlying the progression from NAFLD to atherosclerosis and development of cardiovascular events remain unclear.

The integrity and functional activity of the endothelial monolayer have been shown to play critical roles in atherogenesis [32]. Extensive endothelial cell damage caused by cardiovascular risk factors can result in endothelial cell apoptosis, with subsequent loss of integrity of the endothelium. The traditional view suggests that endothelial cell repair is exclusively mediated by the adjacent endothelial cells. However, a series of basic and clinical studies prompted by the discovery of bone marrow-derived EPCs have provided new insights into these processes and indicate that circulating EPCs play a pivotal role in endothelial cell regeneration. Reduced circulating EPC levels independently predict atherosclerotic disease progression and future cardiovascular events [17], [33], demonstrating an important role of EPCs in endogenous vascular repair and modulation of the clinical course of atherosclerosis and cardiovascular disease. In the current study, we first showed that NAFLD patients had decreased circulating EPC levels and attenuated EPC adhesive function, which implied reduced endothelial repair capacity in patients with NAFLD. This is in agreement with previous studies showing that NAFLD patients had endothelial dysfunction and advanced atherosclerosis [19], [34]. The association between NAFLD and endothelial dysfunction, resulting from impaired vascular repair capacity, may contribute to the higher incidence of cardiovascular events in NAFLD patients [35].

Recent studies emphasize the roles of insulin resistance, oxidative stress and subsequent lipid peroxidation, proinflammatory cytokines, adipokines, and mitochondrial dysfunction in the development and progression of NAFLD. In the current study, patients with NAFLD, a hepatic presentation of insulin resistance [36], were shown to have dysfunctional and decreased circulating EPC levels. The mechanisms to explain the relationship between NAFLD and reduced EPC level and function remain to be determined, but are supposed to be related to insulin resistance and enhanced inflammation in NAFLD patients. Insulin resistance, known to be a cause of endothelial dysfunction, is an important feature of NAFLD. Previous reports showed that decreased levels of circulating EPCs were observed in patients with insulin resistance and metabolic syndrome, and these cases were characterized by low-grade inflammation and increased oxidative stress [16], [37]. Increased levels of reactive oxygen species in NAFLD patients may induce subsequent release of pro-inflammatory cytokines [38], [39], which can compromise endothelial function [40]. Enhanced systemic inflammation, and its association with endothelial dysfunction, has been proven to play a key role in atherogenesis. Clinical studies indicated that patients with NAFLD have enhanced inflammation levels in comparison with the levels in controls [41]. Verma and coworkers showed that recombinant human CRP directly inhibits EPC differentiation, survival, and function, at concentrations known to predict adverse vascular outcomes [42]. Therefore, enhanced inflammation and insulin resistance observed in patients with NAFLD may suppress the levels and function of circulating EPCs and attenuated repair capacity of vasculature.

Reduced EPC levels, which precede the development of manifested cardiovascular disease, have been shown to play a major role in the development of atherothrombosis, which might be due to a decreased capacity to replace damaged or lost endothelial cells. Reduced EPC levels were an independent predictor of increased intima-media thickness of the carotid artery, a strong indicator of subclinical atherosclerotic disease [43]. A recent study showed that NAFLD patients have a greater risk of carotid atherosclerosis, enlarged mean intima-media thickness, and higher prevalence of carotid plaques [30]. Our current results could link the findings of previous studies and provide novel evidence that NAFLD patients have a higher risk of subclinical atherosclerosis. The presence and severity of fatty liver should be carefully monitored as an independent risk factor for cardiovascular diseases. Detection of fatty liver by abdominal sonographic examination may provide valuable information for cardiovascular disease risk assessment.

### Study Limitations

Some limitations should be mentioned in this study. First, the sample size of this study is rather small. We could only adjust for two confounders (metabolic syndrome and uric acid level) in the multivariate analysis because the number of fatty liver patients is relatively small. Further larger, confirmative studies are needed to verify the result. Second, because the fatty liver was diagnosed by abdominal ultrasonography but not by computed tomography or liver biopsy, we could not negate the possibility that only more severe fatty liver cases could be detected, since abdominal ultrasound can detect liver steatosis only when it is greater than 30%. This might bias the result and should be considered a limitation. Third, we enrolled subjects undergoing diagnostic coronary angiography in our study, and it might bias the results. Fourth, we did not check the degree of insulin resistance by homeostasis model assessment for diabetes patients. Fifth, although our data show NAFLD per se influence EPC number and function, NAFLD is indeed strongly associated with metabolic syndrome and diabetes mellitus. In this study, we could not exclude the possibility that metabolic syndrome and diabetes may play a greater role than NAFLD in influencing EPC. Finally, this was a cross-sectional study, in which we identified an association between circulating EPC numbers and presence and severity of NAFLD. Therefore, we could not confirm whether the decrease in circulating EPCs was the cause or the result of NAFLD. Further prospective studies should be arranged to clarify the cause-and-effect relationship and test whether quantification of EPCs levels could provide additional information over the current risk factors to predict future cardiovascular events in NAFLD patients.

In conclusion, this study demonstrated for the first time that NAFLD patients have decreased circulating EPC numbers and adhesive function than those without NAFLD. These findings suggest that attenuated endothelial repair capacity may contribute to atherosclerotic disease progression and enhanced cardiovascular risk in NAFLD patients. NAFLD should be carefully considered as an independent risk factor for cardiovascular diseases.

## Supporting Information

Table S1 **Baseline characteristics of study subjects for culturing of early EPCs (day 7) and late EPC (P3) (8 NAFLD patients and 8 controls).**
(DOC)

Table S2 **Baseline characteristics of study subjects divided NAFLD into 3 groups according to the severity of fatty liver.**
(DOC)

Figure S1 **Characterization of early and late EPC by using Flow Cytometry analysis.**
(TIF)

## References

[1] Adams LA, Angulo P (2005). Recent concepts in non-alcoholic fatty liver disease.. Diabet Med.

[2] Lonardo A, Bellini M, Tartoni P, Tondelli E (1997). The bright liver syndrome. Prevalence and determinants of a “bright” liver echopattern.. Ital J Gastroenterol Hepatol.

[3] el-Hassan AY, Ibrahim EM, al-Mulhim FA, Nabhan AA, Chammas MY (1992). Fatty infiltration of the liver: analysis of prevalence, radiological and clinical features and influence on patient management.. Br J Radiol.

[4] Fan JG, Zhu J, Li XJ, Li L, Dai F (2005). Prevalence of and risk factors for fatty liver in a general population of Shanghai, China.. J Hepatol.

[5] Tarantino G, Saldalamacchia G, Conca P, Arena A (2007). Non-alcoholic fatty liver disease: further expression of the metabolic syndrome.. J Gastroenterol Hepatol.

[6] Kotronen A, Yki-Jarvinen H (2008). Fatty liver: a novel component of the metabolic syndrome.. Arterioscler Thromb Vasc Biol.

[7] Despres JP, Lemieux I, Bergeron J, Pibarot P, Mathieu P (2008). Abdominal obesity and the metabolic syndrome: contribution to global cardiometabolic risk.. Arterioscler Thromb Vasc Biol.

[8] Chiang CH, Huang CC, Chan WL, Chen JW, Leu HB (2010). The severity of non-alcoholic fatty liver disease correlates with high sensitivity C-reactive protein value and is independently associated with increased cardiovascular risk in healthy population.. Clin Biochem.

[9] Sung KC, Ryan MC, Wilson AM (2009). The severity of nonalcoholic fatty liver disease is associated with increased cardiovascular risk in a large cohort of non-obese Asian subjects.. Atherosclerosis.

[10] Bonetti PO, Lerman LO, Lerman A (2003). Endothelial dysfunction: a marker of atherosclerotic risk.. Arterioscler Thromb Vasc Biol.

[11] Verhaar MC, Rabelink TJ (1998). Endothelial function: strategies for early intervention.. Cardiovasc Drugs Ther.

[12] Werner N, Priller J, Laufs U, Endres M, Bohm M (2002). Bone marrow-derived progenitor cells modulate vascular reendothelialization and neointimal formation: effect of 3-hydroxy-3-methylglutaryl coenzyme a reductase inhibition.. Arterioscler Thromb Vasc Biol.

[13] Hill JM, Zalos G, Halcox JP, Schenke WH, Waclawiw MA (2003). Circulating endothelial progenitor cells, vascular function, and cardiovascular risk.. N Engl J Med.

[14] Vasa M, Fichtlscherer S, Aicher A, Adler K, Urbich C (2001). Number and migratory activity of circulating endothelial progenitor cells inversely correlate with risk factors for coronary artery disease.. Circ Res.

[15] Chen JZ, Zhang FR, Tao QM, Wang XX, Zhu JH (2004). Number and activity of endothelial progenitor cells from peripheral blood in patients with hypercholesterolaemia.. Clin Sci (Lond).

[16] Jialal I, Devaraj S, Singh U, Huet BA (2010). Decreased number and impaired functionality of endothelial progenitor cells in subjects with metabolic syndrome: implications for increased cardiovascular risk.. Atherosclerosis.

[17] Werner N, Kosiol S, Schiegl T, Ahlers P, Walenta K (2005). Circulating endothelial progenitor cells and cardiovascular outcomes.. N Engl J Med.

[18] Senturk O, Kocaman O, Hulagu S, Sahin T, Aygun C (2008). Endothelial dysfunction in Turkish patients with non-alcoholic fatty liver disease.. Intern Med J.

[19] Vlachopoulos C, Manesis E, Baou K, Papatheodoridis G, Koskinas J (2010). Increased arterial stiffness and impaired endothelial function in nonalcoholic Fatty liver disease: a pilot study.. Am J Hypertens.

[20] Koh JH, Shin YG, Nam SM, Lee MY, Chung CH (2009). Serum adipocyte fatty acid-binding protein levels are associated with nonalcoholic fatty liver disease in type 2 diabetic patients.. Diabetes Care.

[21] Grundy SM, Brewer HB, Cleeman JI, Smith SC, Lenfant C (2004). Definition of metabolic syndrome: Report of the National Heart, Lung, and Blood Institute/American Heart Association conference on scientific issues related to definition.. Circulation.

[22] Huang PH, Lu TM, Wu TC, Lin FY, Chen YH (2008). Usefulness of combined high-sensitive C-reactive protein and N-terminal-probrain natriuretic peptide for predicting cardiovascular events in patients with suspected coronary artery disease.. Coron Artery Dis.

[23] Huang PH, Chen YH, Tsai HY, Chen JS, Wu TC (2010). Intake of red wine increases the number and functional capacity of circulating endothelial progenitor cells by enhancing nitric oxide bioavailability.. Arterioscler Thromb Vasc Biol.

[24] Huang PH, Huang SS, Chen YH, Lin CP, Chiang KH (2010). Increased circulating CD31+/annexin V+ apoptotic microparticles and decreased circulating endothelial progenitor cell levels in hypertensive patients with microalbuminuria.. J Hypertens.

[25] Fadini GP, Baesso I, Albiero M, Sartore S, Agostini C (2008). Technical notes on endothelial progenitor cells: Ways to escape from the knowledge plateau.. Atherosclerosis.

[26] Huang PH, Chen YH, Chen YL, Wu TC, Chen JW (2007). Vascular endothelial function and circulating endothelial progenitor cells in patients with cardiac syndrome X.. Heart.

[27] Jepsen P, Vilstrup H, Mellemkjaer L, Thulstrup AM, Olsen JH (2003). Prognosis of patients with a diagnosis of fatty liver–a registry-based cohort study.. Hepatogastroenterology.

[28] Rafiq N, Bai C, Fang Y, Srishord M, McCullough A (2009). Long-term follow-up of patients with nonalcoholic fatty liver.. Clin Gastroenterol Hepatol.

[29] Lin YC, Lo HM, Chen JD (2005). Sonographic fatty liver, overweight and ischemic heart disease.. World J Gastroenterol.

[30] Mirbagheri SA, Rashidi A, Abdi S, Saedi D (2007). Liver: an alarm for the heart?. Liver Int.

[31] Brea A, Mosquera D, Martin E, Arizti A, Cordero JL (2005). Nonalcoholic fatty liver disease is associated with carotid atherosclerosis: a case-control study.. Arterioscler Thromb Vasc Biol.

[32] Fuster V, Badimon L, Badimon JJ, Chesebro JH (1992). The pathogenesis of coronary artery disease and the acute coronary syndromes (2).. N Engl J Med.

[33] Rauscher FM, Goldschmidt-Clermont PJ, Davis BH, Wang T, Gregg D (2003). Aging, progenitor cell exhaustion, and atherosclerosis.. Circulation.

[34] Villanova N, Moscatiello S, Ramilli S, Bugianesi E, Magalotti D (2005). Endothelial dysfunction and cardiovascular risk profile in nonalcoholic fatty liver disease.. Hepatology.

[35] Targher G, Day CP, Bonora E (2010). Risk of cardiovascular disease in patients with nonalcoholic fatty liver disease.. N Engl J Med.

[36] Feldstein AE (2010). Novel insights into the pathophysiology of nonalcoholic fatty liver disease.. Semin Liver Dis.

[37] Miller-Kasprzak E, Bogdanski P, Pupek-Musialik D, Jagodzinski PP (2011). Insulin resistance and oxidative stress influence colony-forming unit-endothelial cells capacity in obese patients.. Obesity (Silver Spring).

[38] Pessayre D, Fromenty B (2005). NASH: a mitochondrial disease.. J Hepatol.

[39] Galli A, Svegliati-Baroni G, Ceni E, Milani S, Ridolfi F (2005). Oxidative stress stimulates proliferation and invasiveness of hepatic stellate cells via a MMP2-mediated mechanism.. Hepatology.

[40] Drexler H, Hornig B (1993). Endothelial dysfunction in human disease.. J Mol Cell Cardiol.

[41] Hui JM, Hodge A, Farrell GC, Kench JG, Kriketos A (2004). Beyond insulin resistance in NASH: TNF-alpha or adiponectin?. Hepatology.

[42] Verma S, Kuliszewski MA, Li SH, Szmitko PE, Zucco L (2004). C-reactive protein attenuates endothelial progenitor cell survival, differentiation, and function: further evidence of a mechanistic link between C-reactive protein and cardiovascular disease.. Circulation.

[43] Fadini GP, Coracina A, Baesso I, Agostini C, Tiengo A (2006). Peripheral blood CD34+KDR+ endothelial progenitor cells are determinants of subclinical atherosclerosis in a middle-aged general population.. Stroke.

