# Supplementary material for: Decreased Circulating Endothelial Progenitor Cell Levels and Function in Patients with Nonalcoholic Fatty Liver Disease
Source: PLoS One. 2012 Feb 16;7(2):e31799. doi: 10.1371/journal.pone.0031799 (PMC3280999; doi:10.1371/journal.pone.0031799)
Supplement: Table S1 — Baseline characteristics of study subjects for culturing of early EPCs (day 7) and late EPC (P3) (8 NAFLD patients and 8 controls). (DOC) [file pone.0031799.s001.doc]

**Table S1.** Baseline characteristics of study subjects for culturing of early EPCs (day 7) and late EPC (P3) (8 NAFLD patients and 8 controls).

|  | No fatty Liver  n=8 | Fatty liver  n=8 | P value |
| --- | --- | --- | --- |
| Age (years) | 73.8 ± 8.5 | 74.5 ± 14.7 | 0.903 |
| Male, n (%) | 4 (50) | 4 (50) | 1.000 |
| Hypertension, n(%) | 7 (88) | 7 (88) | 1.000 |
| Diabetes mellitus, n(%) | 3 (38) | 2 (25) | 1.000 |
| Metabolic syndrome, n(%) | 4 (50) | 7 (88) | 0.282 |
| Coronary artery disease, n(%) | 6 (75) | 6 (75) | 1.000 |
| Peripheral artery disease, n(%) | 3 (38) | 2 (25) | 1.000 |
| Chronic kidney disease, n(%) | 3 (38) | 5 (63) | 0.619 |
| Hyperlipidemia, n(%) | 6 (75) | 4 (50) | 0.608 |
| Body mass index (kg/m^2^) | 26.1 ± 5.2 | 24.3 ± 2.5 | 0.394 |
