# Supplementary material for: Decreased Circulating Endothelial Progenitor Cell Levels and Function in Patients with Nonalcoholic Fatty Liver Disease
Source: PLoS One. 2012 Feb 16;7(2):e31799. doi: 10.1371/journal.pone.0031799 (PMC3280999; doi:10.1371/journal.pone.0031799)
Supplement: Table S2 — Baseline characteristics of study subjects divided NAFLD into 3 groups according to the severity of fatty liver. (DOC) [file pone.0031799.s002.doc]

**Table S2.** Baseline characteristics of study subjects divided NAFLD into 3 groups according to the severity of fatty liver.

|  | No FL | Mild FL | Moderate FL | Severe FL | P value |
| --- | --- | --- | --- | --- | --- |
| n | 68 | 17 | 10 | 7 |  |
| Age (years) | 70 ± 13 | 74 ± 13 | 68 ± 16 | 66 ± 18 | 0.991 |
| Male, n (%) | 32 (47) | 9 (53) | 4 (40) | 3 (43) | 0.440 |
| Waist circumference(cm) | 84.4 ± 9.3 | 89.0 ± 7.4 | 86.2 ± 6.1 | 96.7 ± 7.4 | <0.001 |
| BMI (kg/m^2^) | 24.6 ± 4.0 | 27.3 ± 5.7 | 24.7 ± 5.4 | 28.1 ± 3.9 | 0.029 |
| Cholesterol (mg/dL) | 168 ± 48 | 158 ± 34 | 159 ± 44.4 | 209 ± 68.5 | 0.042 |
| LDL-C (mg/dL) | 98 ± 45 | 106 ± 31 | 102 ± 50 | 122 ± 62 | 0.243 |
| HDL-C (mg/dL) | 47 ± 13 | 44 ± 13 | 41 ± 10 | 40 ± 12 | 0.186 |
| Triglyceride (mg/dL) | 119 ± 62 | 145 ± 72 | 132 ± 57 | 166 ± 80 | 0.241 |
| Creatinine (mg/dL) | 1.8 ± 2.3 | 2.2 ± 2.4 | 1.8 ± 2.0 | 1.4 ± 1.3 | 0.813 |
| Total bilirubin (mg/dL) | 0.5 ± 0.2 | 0.5 ± 0.4 | 0.8 ± 0.5 | 0.6 ± 0.3 | 0.270 |
| ALT (U/L) | 23 ± 23 | 28 ± 32 | 42 ± 23 | 43 ± 24 | 0.113 |
| γGT (U/L) | 39 ± 65 | 37 ± 20 | 55 ± 26 | 60 ± 34 | 0.671 |
| Uric acid (mg/dL) | 5.9 ± 1.8 | 8.0 ± 2.6 | 7.5 ± 2.5 | 8.5 ± 2.4 | <0.001 |
| Fasting glucose (mg/dL) | 135 ± 62 | 121 ± 38 | 160 ± 85 | 143 ± 63 | 0.502 |
| HbA1c (%) in DM patients | 6.9 ± 0.6 | 7.1 ± 0.6 | 7.3 ± 0.9 | 6.9 ± 0.8 | 0.433 |
