# Supplementary figures and images for: Decreased Circulating Endothelial Progenitor Cell Levels and Function in Patients with Nonalcoholic Fatty Liver Disease
Source: PLoS One. 2012 Feb 16;7(2):e31799. doi: 10.1371/journal.pone.0031799 (PMC3280999; doi:10.1371/journal.pone.0031799)

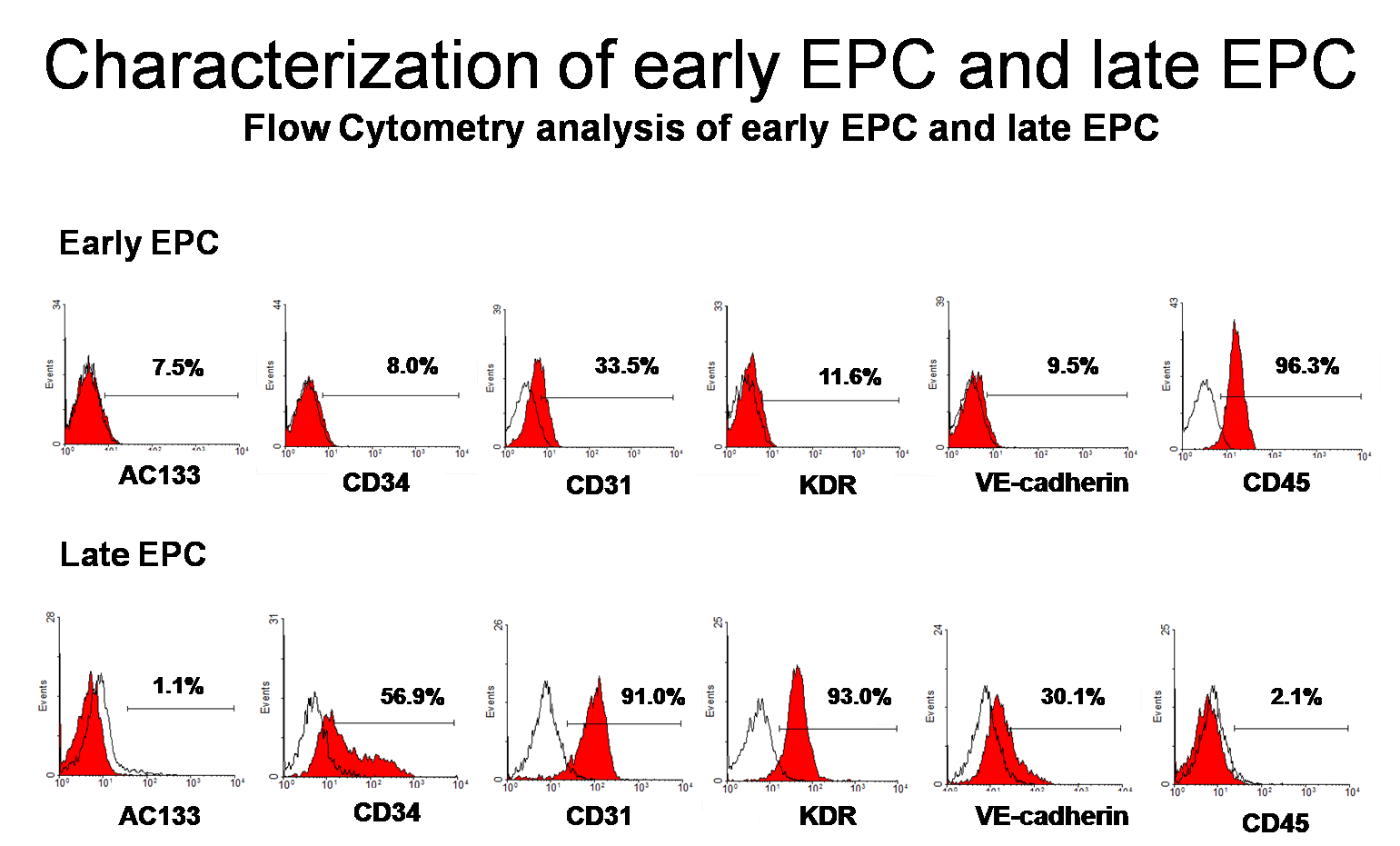

Supplement: Figure S1 — Characterization of early and late EPC by using Flow Cytometry analysis. (TIF) [file pone.0031799.s003.tif]
